# Marital communication skills training to promote marital satisfaction and psychological health during pregnancy: a couple focused approach

**DOI:** 10.1186/s12978-020-0877-4

**Published:** 2020-02-10

**Authors:** Zahra Alipour, Ashraf Kazemi, Gholamreza Kheirabadi, Ahmad-Ali Eslami

**Affiliations:** 1 Student Research Committee, Schools of Nursing and Midwifery, Isfahan University of Medical Sciences, Isfahan, Iran; 2 Nursing and Midwifery Care Research Center, School Of Nursing and Midwifery, Isfahan University of Medical Sciences, Hezarjerib AV, Isfahan, Iran; 3 Behavioral Sciences Research Center, Isfahan University of Medical Sciences, Isfahan, Iran; 4 Department of Health Education and Promotion, Isfahan University of Medical Sciences, Isfahan, Iran

**Keywords:** Anxiety, Depression, Marital satisfaction, Pregnancy, Communication, Couple focused approach

## Abstract

**Background:**

Studies showed that decreased marital satisfaction and increased risk of depression and anxiety were mutually related. Therefore, this study was conducted to evaluate the effect of communication skills training with a couple focused approach on marital satisfaction and psychological symptoms among pregnant women.

**Methods:**

This experimental study was performed on 60 pregnant women with low marital satisfaction who were divided into two groups of intervention and control. The women in the intervention group and their husbands participated in a communication training program based on the couple focused approach. The levels of anxiety, depression, and marital satisfaction were evaluated before and then one and three months after the intervention using valid questionnaires.

**Results:**

The results indicated that after the intervention, compared to the pre-intervention period, the levels of marital satisfaction increased while the levels of depression and anxiety decreased significantly in the intervention group (*p* < 0.05). The comparison of the groups revealed significant differences in the scores of marital satisfaction, depression, and anxiety in the intervention and control groups at the intervals of one month and three months after the intervention (p < 0.05).

**Conclusions:**

The research findings indicated that communication skills training program based on the couple focused approach and an emphasis on the needs of pregnant women during the pregnancy can improve the marital satisfaction and psychological health of pregnant women.

**Trial registration:**

Iranian Registry of Clinical Trials IRCT2017012932264N2, Date of registration: 2017-06-28 Retrospectively registered.

## Plain English summery

Low marital satisfaction and risk of depression and anxiety were mutually related. Therefore, this study was conducted to evaluate the effect of communication skills training with a couple focused approach on marital satisfaction and psychological symptoms among pregnant women. This experimental study was performed on 60 pregnant women with low marital satisfaction. The women in the intervention group and their husbands participated in a communication training program. The levels of anxiety, depression and marital satisfaction were evaluated before, then one month and three months after the intervention using valid questionnaires. The results indicated that after the intervention, compared to the pre-intervention period, the levels of marital satisfaction increased, while the levels of depression and anxiety decreased significantly in the intervention group. The comparison of the groups revealed significant differences in the scores of marital satisfaction, depression, and anxiety in the intervention and control groups at the intervals of one month and three months after the intervention. The research findings indicated that communication skills training program can improve the marital satisfaction and psychological health of pregnant women.

## Background

Physical and psychological changes in women during pregnancy and the transition to the parenthood period pose new challenges and may be followed by poor relationship between the spouses and may reduce the quality of marital relationship [1, 2]. These circumstances can lead to development of psychological disorders in pregnant women, including depression and anxiety [3]. These disorders may have some negative impacts on the outcome of pregnancy. The existing evidence shows that pregnancy depression is associated with an increased risk of preeclampsia and preterm labor as well as low birth weight [4–6]. In addition, the use of Selective Serotonin Reuptake Inhibitors, which is useful in the treatment of pregnancy depression, has recently been reported to increase fetal complications including low birth weight and decreased neonatal Apgar score [7].

Furthermore, antenatal psychological disorders are associated with an increased likelihood of postpartum depression [8]. There is also evidence showing that pregnancy depression and anxiety can decrease the quality of mother-infant communication [9]. Therefore, efforts for modifying the factors that can improve women’s mental health are important in ensuring maternal and child health.

Previous studies showed that a psychological intervention for eliminating anxiety and fear in pregnant women caused by interventional prenatal diagnosis was effective [10]; however, based on the results of some systematic reviews, these studies were not sufficient for giving verdict on the impact of psychological interventions on mental health in women with vulnerable pregnancies [11, 12]. In order to maintain and improve the mental health of pregnant women, we need to increase the ability of couples to resolve their conflicts, after formation of new relationships between partners due to the physical and emotional changes during pregnancy. Evidence suggests that acquiring communication skills can improve the quality of marital relationships and reduce psychological disorders such as depression and anxiety [13].

Factors affecting the psychological health of pregnant women have a great deal of diversity and complexities, and communication skills training may be associated with different results compared to other periods of life. Accordingly, this study was performed to assess the impact of communication skills training on marital satisfaction and levels of depression and anxiety in pregnant women by focusing on the emotional-psychological needs of women during pregnancy.

## Methods

This randomized controlled field trial was conducted with the approval of the Ethics Committee of Isfahan University of Medical Sciences on pregnant women in prenatal care from August 2017 to January 2018 in Isfahan, Iran. The study inclusion criteria were a gestational age less than 24 weeks, the absence of systemic diseases, not taking antidepressants and anti-anxiety drugs, lack of polygyny, and low levels of marital satisfaction. The sample size was considered as 30 subjects for case and control groups with 95% confidence level and test power of 80%. The samples were selected from those referred to Isfahan health centers after obtaining informed consent.

The health centers were selected by random clustering method. Health services in Isfahan are organized by two health care networks. Therefore, two networks were considered as clusters and four health care centers covered by each network were selected randomly. Pregnant women who underwent prenatal care in the health care centers were selected through convenience sampling.

**Fig. 1:**
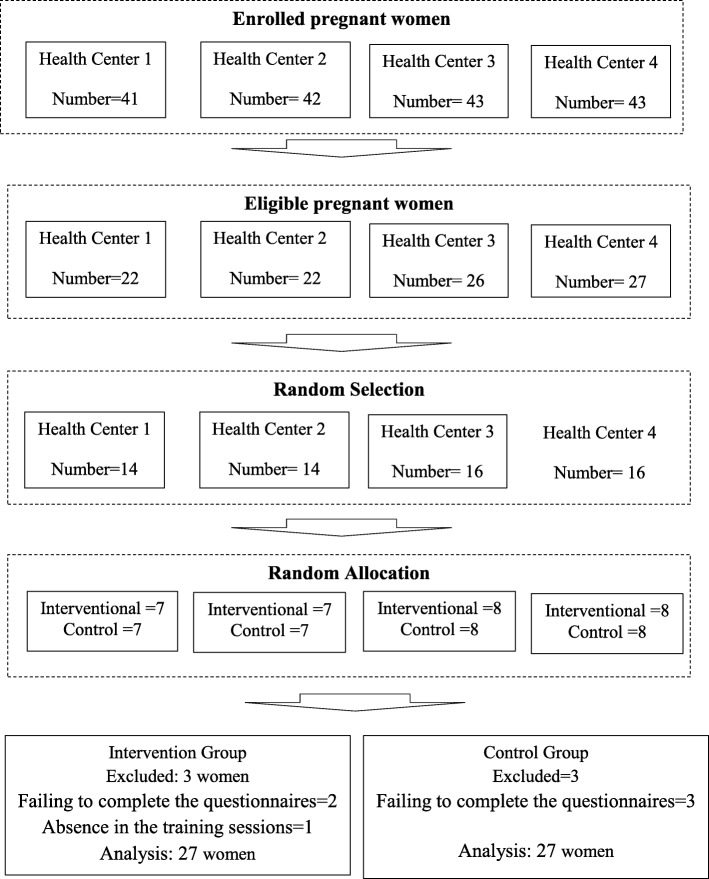
Consort diagram

Initially, 169 pregnant women were enrolled in the study and completed the ENRICH marital satisfaction questionnaire and answered the questions related to the subscales of depression and anxiety of General Health Questionnaire (GHQ). To determine the sensitivity and specificity of marital satisfaction questionnaire and obtain the best cut-off point for marital dissatisfaction, the marital satisfaction scores were used in the agreement with the scores of anxiety and depression scales of the GHQ by applying the “Receiver Operating Characteristic Curve ROC Curve. The cut-off point of 28 was determined based on a sensitivity of 66.1% and specificity of 35.45%. Thus, out of 169 participants, the marital satisfaction score of 97 women was less than 28. Among the 97 eligible women, 60 women were selected randomly and, after recording their demographic characteristics, marital satisfaction questionnaires and sub-scales of depression and anxiety of the GHQ were completed by them. In order to assign the participants randomly to the intervention and control groups, 60 closed envelopes were prepared containing 30 blue and 30 green cards (Fig. 1). The envelopes, together with the file number of the participants, were provided to the prenatal care givers at four centers, and they were asked to give one of the envelopes to the participant who is referred to their ward. The prenatal care providers, therefore, were blinded to the study group. The researcher, then, assigned the blue-carded couples to the intervention group and the green-carded couples to the control group.

After implementing the educational program, the marital satisfaction and levels of depression and anxiety were reevaluated one and three months after the training course.

The data were collected and subsequently analyzed in a blinded method, that is, the prenatal care providers and data analyzer were blinded to the study group. Also, each group was not aware of any other type of intervention.

### Intervention

The communication skills training package with the couple-based intervention was developed based on the documents [14–16] as well as the opinions of experts on psychology, psychiatry and reproductive health. For example, a psychologist believed that to improve the couple’s relationship, the couples’ mutual understanding of each other’s feelings and conditions are necessary factor. In this regard, a reproductive health specialist recommended that psychological changes in women during pregnancy be included in the program content. Both the psychiatrist and the psychologist believed that training mindfulness skills in couples would contribute to the mutual understanding and management of their relationships.

Communication skills training program was conducted in four health centers for couples during 7 sessions. The duration of each session was 2 h and 2 sessions were held each week. 7 to 8 couples from each center participated in the study. The training was delivered as lectures, group discussions and role playing, under the supervision of a specialist in clinical psychology. At the beginning of each session, the skill learned in the previous session was practiced practically for half an hour.

The educational subjects of training sessions were focused on the re-establishment of appropriate communication between partners including the establishment of a good relationship and creation of an atmosphere of trust and security, understanding the changes and psychological needs of pregnant women, the importance of communication skills, psychological health during pregnancy, the description of the differences between men and women, common problems in the family, training of self-awareness and self-care skills, and the assessment of the communication barriers in them.

During training, the problems were described by couples, and the self-awareness strategy training was provided, to be used at an occurrence of interpersonal problems and describing the cycle of awareness and familiarity. Then the five parts of awareness cycle were practiced by the couples, including sensory information, expressing the thoughts, feelings, desires and expectations with respect to the needs and changes of pregnancy [16].

Self-awareness strategies included self-assessment, identification of the strengths and weaknesses, positive thinking, and the development of positive self-image. Activities were designed to empower couples for each of these strategies, and exercises were trained for the identification of emotions and visualization of abilities and desires, as well as awareness of individual characteristics.

During the remaining sessions, the training was focused on the effective marital relationship, barriers to effective communication, describing the types of problems in three categories of thematic problems, own or spouse’s personal problems, and relational problems. Moreover, the skills of the message sender and receiver skills, effective listening skills and speaking skills, identifying factors contributing to active listening, expressing the emotions presented and six speaking skills including showing emotions, expressing demands, outlining the actions, speaking on behalf of oneself, describing the sensory information, expressing opinions with respect to changes of pregnancy were practiced by using the awareness cycle.

The contents of the sixth and seventh sessions included how to identify the conflict and the factors affecting it, couple’s familiarity with effective conflict management, identifying common interests and respecting individual interests, identifying unsuccessful models of conflict resolution, training to discuss the role of unrealistic expectations in the development of conflict, effective conflict management, and teaching skills of stress and anger management. Also, the discussions were summarized to make conclusions and answer the questions. For the couples in the control group, two sessions of childbirth preparation were provided, including familiarity with the exercises during pregnancy, pregnancy and childbirth process, obstetric problems and complications and providing preventive strategies to deal with complications during pregnancy.

Since marital satisfaction was low in the intervention group and because of the moral considerations, the educational pamphlets were also given to the subjects of the control group after the completion of the third phase of the study. The importance of marital relationship improvement for promoting the mental health of the pregnant women had been explained in this pamphlet.

### Tools

To measure the marital satisfaction level, the 47-question form of valid ENRICH questionnaire was used [17]. This questionnaire is designed with four domains including marital satisfaction, sexual satisfaction, conflict resolution and marital communication in a five-item Likert scale (0–4): strongly disagree (0), disagree (1), somehow disagree or agree (2), agree (3), and strongly agree (4). The questionnaire sum score ranged from 0 to 188 points and the higher scores indicate the higher marital satisfaction.

To measure depression and anxiety levels, the subscales of anxiety and depression of valid General Health Questionnaire [18] were used. Each of the depression and anxiety subscales consists of seven items. This tool is designed based on 4-item Likert scale (0–3): strongly disagree (0), disagree (1), agree (2), and strongly agree (4). The questionnaire sum score ranged from 0 to 21 points. The higher scores indicate the higher depression and anxiety levels.

### Statistical analysis

The data obtained from the study were analyzed using SPSS 19 software and statistical methods of the independent t-test, pair t test, chi-square, repeated measures analysis of variance (RMANOVA), and Pearson correlation coefficient. Also, the data normality was evaluated through the Kolmogorov-Smirnov test.

## Results

Of the 60 participants of the study, 6 subjects were excluded from the study that was due to their failure in completing the questionnaires and not attending more than 30% of the training sessions in the intervention group (Fig. 1). Then, the analysis was performed on data from 54 participants with an average age of 29.5 years. The Kolmogorov test results showed that the scores of depression, anxiety, and marital satisfaction variables had a normal distribution. Comparing the background characteristics and the scores of marital satisfaction, anxiety, and depression before the intervention revealed no significant differences (Table 1). Comparison of the means scores of marital satisfaction, depression, and anxiety in three intervals of before, one month and three months after the intervention in both groups are given in Table 2. The level of depression and anxiety three month after intervention was lower (*p* = .001) and the marital satisfaction (*p* = .003) was higher in the intervention group than in the control group. The results showed the impact of group in the level of depression (*p* = .03), anxiety (*p* < .001) and marital satisfaction (*p* = .004) were significant. Also, the results showed that during the study follow-ups in the intervention group, significant changes in the levels of anxiety, depression and marital satisfaction (p < .001) occurred (Table 3).

**Table 1:** Comparison of baseline characteristics in study groups

	Intervention Group (*n* = 27)	Control Group (n = 27)
	Mean (SD) or Number (%)	Mean (SD) or Number (%)
Age of husband	33.5 (4.2)	33.5 (5.1)
Age of women	29.1 (4.3)	29.4 (4.5)
Gestational age	18.7 (6.8)	21.4 (4.1)
Length of marriage	4.6 (3.8)	5.4 (4.2)
Education level of husband (%)		
High school or less	16 (59.2)	15 (55.5)
More than high school	11 (40.8)	12 (44.5)
Education level of women (%)		
High school or less	10 (37.1)	15 (55.5)
More than high school	17 (62.9)	12 (44.5)
Occupational status of women (%)		
Employed	4 (14.8)	5 (18.5)
Economic level (%)		
Low	1 (3.7)	1 (3.7)
Medium	19 (70.4)	20 (74.1)
High	7 (25.9)	6 (22.2)
Parity (%)		
Primparity	19 (70.4)	16 (59.3)
Multiparty	8 (29.6)	11 (40.7)
History of abortion (%)	3 (11.1)	5 (18.5)
Obstetric complications (%)	3 (11.1)	2 (7.4)

*Abbreviations*: *SD* standard deviation, *sig* significance, *ns*. not significant

**Table 2:** Comparison of anxiety, depression, and marital satisfaction scores between two groups by three times

	Mean (SD)			Time/ Group			
	Intervention Group (*n* = 27)	Control Group (*n* = 27)	Sig^a^	F^b^	Sig^b^	F^c^	Sig^c^
Anxiety							
At Intake	8.33 (4.67)	7.74 (4.18)	ns	14.21	<.001	4.94	.03
After 1 month	4.29 (2.35)	7.22 (4.90)	.007				
After 3 month	3.25 (2.87)	6.77 (3.96)	.001				
Depression							
At Intake	2.88 (2.75)	4.40 (5.37)	ns	4.88	.03	7.14	.01
After 1 month	1.66 (1.86)	3.14 (4.07)	ns				
After 3 month	0.74 (1.16)	3.81 (4.48)	.001				
Marital satisfaction							
At Intake	155.25 (21.09)	152.55 (22.27)	ns	9.30	.004	4.93	.04
After 1 month	170.00 (15.98)	159.44 (21.80)	<.05				
After 3 month	177.18 (17.33)	160.22 (23.02)	.003				

*Abbreviations*: *SD* standard deviation, *sig* significance, *ns* not significant

^a^: Results of t test

^b^: Results of repeated measures analysis of variance (RMANOVA)

^c^ test of within subject effect

^b^ test of between subject effect

**Table 3:** The results of pair t test for anxiety, depression, and marital satisfaction

		Sig		
Group	Time	Anxiety	Depression	Marital satisfaction
Intervention Group				
At intake	After 1 month	<.001	<.001	<.001
	After 3 month	<.001	.001	<.001
After 1 month	After 3 month	.04	.02	.006
Control Group				
At intake	After 1 month	ns	ns	.04
	After 3 month	ns	ns	ns
After 1 month	After 3 month	ns	ns	ns

*Abbreviation*: *G* group

Results showed a significant inverse correlation between the level of marital satisfaction and anxiety and depression scores before, 2 and 3 months after the intervention (Table 4).

**Table 4:** The Correlation between marital satisfaction, depression and anxiety before and one month and three months after the intervention

	1	2	3	4	5	6	7	8	9
1-Anxiety At Intake	–								
2-Depression At Intake	.45^**^	–							
3-Marital Satisfaction At Intake	−.38**	−.36**	–						
4-Anxiety At 2th Section	.54**	.35*	−.19	–					
5- Depression At 2th Section	.30*	.29*	−.14	.57**	–				
6- Marital Satisfaction At 2th Section	−.26	−.40**	.72**	−.32*	−.29*	–			
7- Anxiety At 3th Section	.42**	.11	−.30	.50**	.17	−.37**	–		
8- Depression At 3th Section	.35**	.78**	−.20	.42**	.21	−.26	.26	–	
9- Marital Satisfaction At 3th Section	−.33*	−.40**	.64**	−.38**	−.22	.81**	−.49**	−.42**	–

* *P* < 0.05, ** *p* < 0.01

## Discussion

The aim of this study was to evaluate the effect of teaching communication skills on marital satisfaction and psychological health of pregnant women with low levels of marital satisfaction. The present study indicated that the efforts made to increase women’s communication skills would be associated with their improved psychological health and increased marital satisfaction. Many studies have shown that communication skill training is effective in reducing marital conflicts [19, 20] and improving the communication between couples [16], and improves the couples’ psychological health [13, 21]. During pregnancy, due to physical and emotional changes in women, abnormal relationships may be experienced by the couples [2]; however, this study confirmed that during pregnancy, like other periods of life, communication skills training for women can improve such communications and relationships. Meanwhile, the results of this study showed that although the effect of communication skills training on marital satisfaction was greater than the baseline up to three months after the intervention, the marital satisfaction within one to three months after the intervention did not increase, while it is expected that acquiring communication skills over time would be influenced by further impacts of improved relationships in this period. This finding suggests that efforts to improve communication skills during pregnancy require supplemental plans to stabilize the effect of the program on couple’s communication skills. In addition, no increase in the marital satisfaction between one and three months after the intervention cannot be attributed to increased gestational age since in the control group, the level of marital satisfaction three months later had no significant difference with either the arrival time or a month later.

Another finding of the study suggested that the training program of communication skills is associated with the improved psychological health of pregnant women so that the level of depression in the intervention group fell following the communication skills promotion. This finding reflects that programs designed to increase communication skills can reduce the level of depression in women lacking a good marital quality.

Contrary to this finding, the available studies’ results have shown that the levels of anxiety and depression in women decrease in the mid-pregnancy [22, 23]. Therefore, the present results show that women with marital dissatisfaction during pregnancy would not experience the decline in the level of depression, but in women who acquire communication skills, the level of depression will decrease.

Another research result, indicating a correlation between the level of marital satisfaction and the level of depression, confirms this explanation. Systematic review studies have also shown that other programs effective in marital satisfaction, such as behavioral program and multimodal cognitive behavioral programs are also associated with reduced levels of anxiety and depression [24]. Assessing the impact of cognitive behavioral therapy intervention on reducing depression during pregnancy and after delivery as well as the results of this study consistently have shown that the development of communication skills has a positive effect on marital satisfaction and improving psychological disorders [25]. Another study also reported that marital relations training in the last trimester of pregnancy is associated with enhanced marital relations and improved mental disorders [26].

The impact of the training program on management of relationships and psychological health of mothers has also been reported with an emphasis on features and changes during the pregnancy, changes in the relations of couples and management of couple’s relationships through creating intimacy and empathy as well as strategies to cope with anxiety and depression [27].

Enhancing communication skills, in addition to improving interpersonal relationships, of couples through the promotion of conflict resolution skills may also affect the coping styles of people, and thus, improves the marital satisfaction [14] and psychological health [15].

In addition to the effect of communication skills training program on levels of depression, the effectiveness of this program on reducing anxiety was another finding of this study. The results indicated that simultaneous with decreased levels of depression, the levels of anxiety decreased even more than the depression following the acquisition of communication skills in couples, which suggests that increased intimacy between couples and improved relationships following acquisition of communication skills can also be accompanied with a reduction in anxiety.

The strength of the study was couple focused approach of the intervention; however, if marital satisfaction had been assessed in men, the interaction between the couple’s marital satisfaction and pregnant women’s mental health would also be detectable. Additionally, the interrelationship between the couples and their behaviors was not assessed, which might limit the prediction of the continuity of the intervention effect. In addition, defined gender roles in different societies may affect couple relationships. Therefore, the effect of the communication skills training intervention to improve the mental health in pregnant women in different communities should be evaluated.

## Conclusion

The evidence provided that the lack of communication skills may be grounds for reducing the quality of couples’ relationships; however, enhancing this quality through communication skills training during pregnancy can lead to enhancing women’s psychological health during pregnancy.

## Data Availability

The datasets used during the current study are available from the corresponding author on reasonable request.

## Abbreviations

G: Group
ns: Not significant
RMANOVA: Repeated measures analysis of variance
SD: Standard deviation
Sig: Significance
